# Case of Fistula Lacrymalis

**Published:** 1804-06-01

**Authors:** H. Crump

**Affiliations:** Albrighton


### Case of Fistula Lacrymalis.

*Communicated by* Mr. H. Crump, *of Albrighton.*

THE subject of the disease was a young woman of tolerable good health, but rather disposed to strumous affections, ætat 19; she had for three months laboured under a painful affection of the right eye, with slight inflammation, and a particular fullness of both eye-lids. The tears now and then passed over the cheek, which she could not account for, and then a distressing heat and dryness of the nostril on the right side came on, which was soon relieved when the tears took their proper channel.

I directed some leeches to be applied under the lower eye-lid, and the use of a saturnine lotion, c. tinct. opii, and a poultice at night made with crumbs of bread moistened with a strong decoction of poppies. This treatment was persisted in for a fortnight; the pain had entirely left her, as well as the inflammation and thickening of the eye-lids. The leeches were applied three times. (The *tarsus* on both lids were the parts most inflamed, especially at the inner canthus and *puncta* lacrymalia.) She now considered herself well, and I did not see her again for a month, when I was called to her to treat the same complaint. I found her under very different circumstances, the disease much aggravated; instead of an occasional flow of tears over the cheek, they were constantly oozing from the puncta and dribling down the cheek. Pain in the eye, inflammation of the lids, and thickening of them had returned, with heat and dryness of the nostril. The lacrymal sac now, was much distended, so much so that the tumour was evident at some distance; pressure soon emptied it, the tears escaping at the puncta, but in a very short time it filled again.

Topical bleeding was again used, with the same application, varying them a little; however, she reaped no benefit from the bleeding, nor any of the applications used; and, as her constitution began to suffer from the constant source of irritation being kept up, I gave her decoct. angustur. c. acid. nitros. which she benefitted much by; the pain was relieved by the occasional exhibition of opiates. I now thought the case promised fair for the success of an operation, especially as the obstruction in the canalâ nasali had recently taken place, which induced me to attempt the cure in a way I have seen Mr. Astley Cooper practice (when a pupil of his) in Guy's Hospital, and which I shall now attempt to describe. A common sized blunt probe being bent, giving it such a course as to take the direction of the lacrymal canal, was cautiously introduced under the inferior turbinated bone, passing it on till the head of the probe became fixed in the duct, using no force till I was sure the probe was in this situation, which is very soon known, especially by one that has ever introduced it; I then depress the point of the probe, in order to elevate the blunt end, and if the obstruction is not great it will pass forward to the extremity of the duct, and even into the sac itself, which was the case with my patient. When I found the probe had reached the sac, after remaining a minute, I withdrew it; the sac then, which was before distended, forming a large tumour, was soon closed, the contents taking the course of the duct. I continued introducing the probe once a day for a fortnight, which I am happy to say completed the cure. It may be necessary to observe, that after the five first introductions of the probe a free hæmorrhagy followed; I then, in a few days, observed it covered with pus, and at length mucus only, when I considered the canal perfect. My patient has now been well six months, and has never suffered the least inconvenience in the part since, nor did she take any medicine after the first introduction of the probe.

I would recommend Students in the dissecting-room to practice the introduction of the probe in the above way, on the dead subject; a few trials is sufficient to make any one expert at it, having previously dissected the parts, and thus become acquainted with the direction of the canal.

I shall, in a short time, trouble you with an account of a case of strangulated scrotal hernia, which I operated upon with success, after a stricture upon the intestine for six days; during which time, distressing symptoms were present.

*May* 11, 1804.

